# Effect of predictive nursing based on risk early warning system on patients with acute respiratory failure in Intensive Care Unit

**DOI:** 10.12669/pjms.40.8.9506

**Published:** 2024-09

**Authors:** Mimi Pan, Lizhong Zhang

**Affiliations:** 1 Mimi Pan Department of Emergency, Wenzhou People’s Hospital, Wenzhou City, Zhejiang Province 325000, P.R. China; 2 Lizhong Zhang Department of Emergency, Wenzhou People’s Hospital, Wenzhou City, Zhejiang Province 325000, P.R. China

**Keywords:** Predictive nursing, Acute Respiratory failure, ICU

## Abstract

**Background & Objective:**

Acute respiratory failure (ARF) is a life-threatening condition that necessitates intensive care and often results in high morbidity and mortality. Predictive nursing, combined with a risk early warning system, offers a proactive approach to patient care that could potentially improve outcomes in patients with ARF. However, the efficiency of this approach in intensive care settings is still unclear. This study aimed to analyze the effect of predictive nursing based on risk early warning system in patients with acute respiratory failure in intensive care unit (ICU) setting.

**Methods:**

A retrospective cohort study included records of 368 patients admitted to ICU of a tertiary care hospital due to ARF from January 2021 to January 2023. Patients were divided into two groups based on the received care: standard care (control group, n=197) and predictive nursing care based on a risk early warning system (observation group, n=171). Data on demographics, clinical characteristics, complications, Acute Physiology, Age and Chronic Health Evaluation-II (APACHE-II) and Sequential Organ Failure Assessment (SOFA) scores, and duration of hospitalization were collected and analyzed.

**Results:**

The observation group exhibited significantly lower incidence of complications related to ventilator use and shorter durations of mechanical ventilation, ICU stay, and total hospitalization compared to the control group (p<0.001). Furthermore, patients in the observation group had significantly lower APACHE-II and SOFA scores and blood lactate levels at both one week and two weeks post-intervention.

**Conclusion:**

Predictive nursing care based on a risk early warning system significantly improved clinical outcomes and reduced mortality rates in ICU patients with ARF. The results underscore the potential of integrating predictive nursing care into routine practice, thereby transforming the care paradigm for ICU patients with ARF. Future research should explore the applicability of predictive nursing for other clinical conditions and in various healthcare settings.

## INTRODUCTION

Acute respiratory failure (ARF) is characterized by an inability of the respiratory system to meet the body’s oxygen needs or eliminate carbon dioxide. It also accounts for a large share of hospitalizations in intensive care units globally, thus requiring immediate medical attention and best practice care.^1^,^2^ However, despite recent advances in medical technology and care models, ARF still presents with significant morbidity and mortality rates, which are also associated with huge costs of health systems.^3^ It leads to prolonged ICU stays, increased risk of complications and eventually high risk of mortality.^2^,^3^

Predictive nursing is one such innovative approach supported by principles of identifying risks as well as acting in advance that has potential to revolutionize ICU care for patients with ARF.^4^ In contrast, traditional nursing care involves reactive measures taken after complications have occurred.^5^ Predictive nursing on other hand employs a proactive approach using an early warning system to anticipate problems before they occur.^4^

Predictive nursing uses an early warning system which assists in analyzing patient data obtained from various sources using algorithms, thereby helping to reduce risks for patients with ARF, while also improving healthcare outcomes.^4^,^6^ This can help nurses detect any possible complications among patients suffering from ARFs even before they get worse hence facilitating timely intervention. However, the effectiveness of predictive nursing and the role of early warning systems in managing patients with ARF in ICUs remain unclear. Our study aimed to assess the outcomes of patients with ARF who received predictive nursing based on a risk early warning system in the ICU setting, and to compare them to the outcomes of patients who received a standard nursing care.

## METHODS

This study followed a retrospective cohort design to explore the impact of predictive nursing care based on a risk early warning system for patients with acute respiratory failure in the ICU. The cohort consisted of 368 patients aged ≥18 years admitted to ICU of our tertiary care hospital for ARF between January 2021 and January 2023. Patients were divided into two groups based on the nursing care they received during their ICU stay. Patients in the observation group received predictive nursing based on a risk early warning system, whereas the control group received routine nursing care. Allocation to the control or observation group was not randomized; it was based on the care protocol at the time of their admission.

### Ethical Approval:

The ethics committee of Wenzhou People’s Hospital approved this study on December 13^th^, 2023, with the No. KY-2023-374.

### Inclusion criteria:

Patients diagnosed with ARF^7^; patients able to communicate effectively and willing to have their data included in the study.

### Exclusion criteria:

Patients with severe cognitive impairments, psychiatric disorders, or other significant comorbidities that could have influenced the primary outcomes; patients with an ICU stay less than 24 hours or readmitted to the ICU within the same hospital stay.

### Intervention Details:

The intervention consisted of predictive nursing care provided by two nursing personnel, incorporating an early risk warning system designed to preempt potential complications in patients with acute respiratory failure. The system continuously monitored a variety of physiological parameters, such as heart rate, blood pressure, oxygen saturation, and respiratory rate, along with clinical parameters including laboratory results and chest radiography findings. Early warning system used an algorithm-based risk assessment model which evaluated patient vitals, lab results, and other pertinent clinical data, alerting healthcare providers about potential complications or deteriorations in patient status. The aim of this intervention was to reduce the severity of illness, incidence of complications, and hospitalization duration in patients with ARF.

### Control group details:

The control group consisted of the patients receiving routine nursing care during the ICU stay for ARF. This standard care did not include use of early risk warning system and based on usual nursing protocols followed in ICU at the admission time.

### Data Collection:

Data were extracted from electronic health records. Data extraction was performed by trained clinical researchers who were blinded to the purpose of the study to reduce bias. Variables recorded included demographic data such as gender, age, body mass index (BMI), smoking habits, alcohol consumption, and the presence of underlying diseases. Clinical information including acute physiology and chronic health evaluation-II (APACHE-II) scores, sepsis related organ failure assessment (SOFA) scores, blood lactate levels, incidence of complications, duration of mechanical ventilation, ICU stay time, and total hospitalization time were also collected. These parameters were recorded at admission and then daily until ICU discharge. The main outcomes were recorded at baseline, one week and two weeks after the intervention.

### Acute Physiology and Chronic Health Evaluation-II (APACHE-II):

The APACHE-II scoring system is a severity-of-disease classification system.^8^ It is one of the most commonly used ICU scoring systems globally, with a higher score indicating a more severe condition and a higher risk of death.

### Sepsis-Related Organ Failure Assessment (SOFA):

The SOFA score is a scoring system used to track patient’s status during the stay in an ICU.^9^ It determines the extent of organ function or rate of failure, with a higher score indicating a more severe condition and higher mortality risk.

### Blood Lactate Levels:

Blood lactate levels were measured as a marker of tissue hypoperfusion and oxygen debt.^10^ Elevated levels often indicate severe illness and are associated with increased morbidity and mortality in ICU patients.

### Data Analysis:

Data was compiled into Microsoft Excel and analyzed using STATA software, version-17. Shapiro-Wilk test was utilized to assess the normality of data. Descriptive statistics were represented as means ± standard deviations (SD) for normally distributed continuous variables or as medians with interquartile ranges (IQR) for non-normally distributed data. Independent t-tests or Mann-Whitney U tests, as appropriate, were used to compare continuous variables between the observation and control groups. Categorical variables were reported as numbers and percentages. Chi-square tests or Fisher’s exact tests were used to analyze differences between the two groups for categorical variables.

Univariate analysis was conducted initially to identify potential factors associated with outcomes. Variables with a p-value less than 0.2 in the univariate analysis were included in a multivariate logistic regression model to identify independent predictors of outcomes. A p-value less than 0.05 was considered statistically significant. All p-values mentioned are two-tailed.

## RESULTS

In the present study, we retrospectively evaluated clinical records of 368 ICU patients with diverse primary diseases, including pneumonia, bronchial asthma, chronic obstructive pulmonary disease, and other conditions, with a mean age of 61.23 (8.39) years. A control group consisted of 197 patients who received a standard nursing care, and an observation group contained 171 patients who received predictive nursing based on a risk early warning system.

As summarized in Table-I, mean ages for the control and the observation groups were 60.96 and 61.54 years, respectively, and the gender distribution was comparable (58.4% and 52.6% males in the control group and the observation group, respectively). The groups were not substantially different with respect to BMI, or the length of their disease. Both groups also had comparable primary disease classifications. Mean APACHE-II and SOFA scores, markers of the severity of critical illness and multiple organ dysfunction, respectively, were also similar across the groups (Table-I).

**Table-I:** Baseline characteristics of the study participants.

Characteristics	Control Group (n=197)	Observation Group (n=171)	Total (n=368)
Age (Years)	60.96 (7.69)	61.54 (9.15)	61.23 (8.39)
***Gender***			
- Male	115 (58.4%)	90 (52.6%)	205 (55.7%)
- Female	82 (41.6%)	81 (47.4%)	163 (44.3%)
BMI (kg/m^2)	23.82 (2.91)	23.41 (2.96)	23.63 (2.93)
Course of Disease (Days)	5.91 (2.28)	6.23 (2.39)	6.06 (2.33)
***Primary Disease***			
- Pneumonia	101 (51.3%)	74 (43.3%)	175 (47.6%)
- Bronchial Asthma	57 (28.9%)	50 (29.2%)	107 (29.1%)
- Chronic Obstructive Pulmonary Disease	26 (13.2%)	27 (15.8%)	53 (14.4%)
- Others	13 (6.6%)	20 (11.7%)	33 (9.0%)
APACHE-II	22.97 (4.07)	23.60 (4.59)	23.26 (4.32)
SOFA	4.24 (1.16)	4.05 (1.09)	4.15 (1.13)
Blood lactate levels (mmol/L)	22.67 (3.89)	23.19 (3.29)	22.91 (3.63)

Values are presented as mean (standard deviation) for continuous variables and as frequency (percentage) for categorical variables.

In terms of the incidence of complications (Table-II), there was a significantly lower incidence of ventilator application (p=0.006), reintubation, and unsuccessful one-time weaning in the observation group compared to the control group (p<0.001).

**Table-II:** Incidence of complications between observation and control group.

Characteristics	Total (n=368)	Observation Group (n=171)	Control Group (n=197)	Chi square statistic	p-value
***Ventilator application***					
None	305	154 (50.49%)	151 (49.51%)	12.55	0.006
Ventilator associated pneumonia	34	9 (26.47%)	25 (73.53%)		
Ventilator associated lung injury	19	4 (21.05%)	15 (78.95%)		
Excessive dependence on ventilators	10	4 (40%)	6 (60%)		
***Reintubation***					
No	333	167 (50.15%)	166 (49.85%)	19.09	<0.001
Yes	35	4 (11.43%)	31 (88.57%)		
***Successful one-time weaning***					
No	71	20 (28.17%)	51 (71.83%)	11.84	0.001
Yes	297	151 (50.84%)	146 (49.16%)		

As shown in Table-III, compared to standard nursing care, predictive nursing based on a risk early warning system was associated with shorter hospitalization (13.32 vs 15.58 days in the control group), shorter ICU stay (7.38 vs 8.85 days), and lower mechanical ventilation duration (6.05 vs 7.18 days). All these differences were statistically significant (p<0.001).

**Table-III:** Comparison of hospitalization, ICU stay and mechanical ventilation duration between observation and control group (n=368).

Characteristics	Observation Group (n=171)	Control Group (n=197)	t test statistic	P-value
Mechanical ventilation time (days)	6.05 ± 2.06	7.18 ± 2.17	5.11	<0.001
ICU stay time (days)	7.38 ± 2.11	8.85 ± 2.15	6.60	<0.001
Hospitalization time (days)	13.32 ± 1.99	15.58 ± 2.13	10.52	<0.001

Comparison of clinical scores and blood lactate levels (Table-IV) showed that the observation group had consistently lower values at both one week and two weeks post-intervention. APACHE-II scores were lower in the observation group after one week (17.96 vs 19.21, p=0.0040) and two weeks (13.63 vs 15.14, p=0.0003). Similarly, SOFA scores were also reduced in the observation group after 1-week (3.08 vs 3.65, p<0.0001) and two weeks (2.58 vs 3.19, p<0.0001). Blood lactate levels also decreased significantly in the observation group compared to the control group at both time points (16.79 vs 18.69 mmol/L at one week and 13.38 vs 16.29 mmol/L at two weeks, both p<0.0001).

**Table-IV:** Comparison of APACHE-II, SOFA and blood lactate levels between observation and control group (1-week and 2-weeks after intervention) (n=368).

Measurement	Group	N	Mean	Std. Err.	Std. Dev.	95% CI (Low)	95% CI (High)	t-test Statistic	P-value
APACHE After 1 week of intervention	Control	197	19.20812	.2796893	3.925627	18.65653	19.75971	2.8972	0.004
	Observation	171	17.95906	.3321296	4.343158	17.30343	18.61469		
APACHE After 2 weeks of intervention	Control	197	15.14213	.2720643	3.818604	14.60558	15.67868	3.6246	0.0003
	Observation	171	13.62573	.3215699	4.205072	12.99095	14.26052		
SOFA After 1 week of intervention	Control	197	3.649746	.0763077	1.07103	3.499257	3.800236	5.4096	<0.0001
	Observation	171	3.076023	.0723129	.9456143	2.933276	3.21877		
SOFA After 2 weeks of intervention	Control	197	3.192893	.0577268	.8102344	3.079048	3.306739	7.7894	<0.0001
	Observation	171	2.584795	.0509694	.6665118	2.484181	2.68541		
Blood lactate After 1 week of intervention	Control	197	18.68528	.2705095	3.796781	18.1518	19.21876	5.1123	<0.0001
	Observation	171	16.79415	.2460024	3.216899	16.30854	17.27976		
Blood lactate After 2 weeks of intervention	Control	197	16.29137	.264036	3.705921	15.77065	16.81209	8.1295	<0.0001
	Observation	171	13.37602	.2358388	3.083993	12.91047			

There were significant differences in APACHE-II, SOFA, and blood lactate levels when comparing values before the intervention to those at one week and two weeks post-intervention. Table-V. All p-values were less than 0.0001, highlighting that the intervention had a substantial, positive effect on these parameters over time.

**Table-V:** Comparison of APACHE-II, SOFA and blood lactate levels before and after the intervention in both the groups (n=368).

Paired t-tests results between measurements	Mean difference	Std. Err. of difference	t-test statistic	p-value
APACHE: Before intervention vs After 1 week	4.633152	.0711385	65.1286	<0.0001
APACHE: Before intervention vs After 2 weeks	8.82337	.0870545	101.3546	<0.0001
APACHE: After 1 week vs After 2 weeks	4.190217	.0373776	112.1050	<0.0001
SOFA: Before intervention vs After 1 week	.7663043	.0260859	29.3762	<0.0001
SOFA: Before intervention vs After 2 weeks	1.23913	.0377983	32.7827	<0.0001
SOFA: After 1 week vs After 2 weeks	.4728261	.0260612	18.1429	<0.0001
Blood lactate: Before vs After 1 week	5.104076	.0803217	63.5454	<0.0001
Blood lactate: Before vs After 2 weeks	6.151324	.0773617	79.5297	<0.0001
Blood lactate: After 1 week vs After 2 weeks	1.047248	.0240935	43.4645	<0.0001

The p-values for all the paired t-tests are less than 0.0001, indicating that the differences in means between the paired measurements are statistically significant at a 0.01% significance level.

## DISCUSSION

This study aimed to assess the impact of predictive nursing care based on a risk early warning system for patients with acute respiratory failure in the ICU setting. Our findings indicate that predictive nursing care is associated with lower incidence and duration of ventilation, lower rates of reintubation and unsuccessful one-time weaning, and shorter hospitalization and ICU stay. Additionally, patients who received this method of nursing had better overall outcomes and metabolic status as indicated by lower SOFA and APACHE-II scores and decreased blood lactate levels. A crucial component of the predictive nursing care approach evaluated in this study was the use of an early warning system to alert healthcare providers about potential complications or deteriorations in patient status. Early warning systems have been recognized as effective tools to improve patient outcomes by facilitating early intervention in a variety of healthcare settings.^11^-^14^000 patients were included in the analysis. Seven key findings were identified, the impact of NEWS on: (a Our results provide further support for the benefits of early warning systems, particularly in the management of ARF in the ICU setting.

In the context of our study, the risk early warning system acted as an intermediary between healthcare practitioners and a complex medical data. The system’s ability to process a wide range of physiological and clinical parameters and translate them into actionable insights marks an exciting advancement in the field of health informatics.^15^ This reinforces the increasing role of technology in enhancing healthcare outcomes and the critical need for healthcare practitioners to be well-versed with digital health tools.^15^,^16^ Employing such systems will lead to a higher standard of care.

The results of our study concur with the overall tendency in healthcare towards more personalized and predictive care models.^4^ In contrast to traditional “one size fits all” approach to healthcare, personalized medicine promises better treatment efficacy and patient outcomes.^17^,^18^ This study extends this idea to nursing care field and demonstrates the possible advantages of predictive nursing care for managing ARF in ICU as seen from significantly reduced needs for interventions such as ventilation, reintubation, and shorter lengths of stay in hospital and ICU.

It is also important to emphasize the transformative potential of predictive nursing on the nursing profession. The change from reactive to proactive patient management increases job satisfaction by enabling nurses to predict, interpret and meet patients’ needs more effectively. In addition, data driven approach provides objective support for decision making processes that can reduce cognitive load on health professionals thus enhance quality of nursing care in general.^15^,^16^ Hence, this intervention not only improves the patient outcome, it may also hold relevance on a nurse’s work environment and job satisfaction, which points towards win-win potential as an innovation within healthcare.

Moreover, our findings offer important insights into the value of predictive nursing care among patients with ARF, which is a complex life-threatening condition responsible for various ICU admissions. Despite recent developments in medical technology and care models, ARF remains a major cause of morbidity/mortality.^19^,^20^ Our results suggest that predictive nursing care could be pivotal in improving patient outcomes concerning ARF, thereby having significant implications on ICU care.

### Limitations:

This is a retrospective study, which can be subject to various forms of bias. For example, there may have been differences in patient characteristics or treatment practices that we were unable to control for, that could have influenced our results. Furthermore, the study was conducted at a single hospital, which may limit the generalizability of our findings to other settings.

The effects of predictive nursing may differ in other conditions or healthcare settings. Thus, future research should examine its applicability and effectiveness across a variety of diseases and care environments. Equally, the psychological impact of predictive care on patients and their families is another aspect worth exploring. An early warning system could potentially create anxiety for patients and families if not managed properly. In addition, our study also opens several avenues for future research that are exciting. For example, subsequent studies may delve into the mechanisms through which predictive nursing care enhances patient outcomes. Similarly, additional investigation might focus on how to best integrate predictive nursing care into existing ICU care models. Also, these systems could become more sophisticated and include a greater range of patient data such as genomic information or data from wearable devices and they can employ an advanced machine learning algorithm so as to predict accurately the clinical outcome of patients.

## CONCLUSION

Our study indicates that predictive nursing care based on a risk early warning system may improve patient outcomes in ICU patients with ARF. These findings could have significant implications for ICU care, highlighting the value of predictive nursing care as a key component of personalized medicine. It is therefore essential to constantly evaluate and refine these approaches, as healthcare landscape moves toward more personalized and predictive care models, aiming at improving patient outcomes and quality of care.

### Authors’ Contributions:

**MP:** Conceived and designed the study.

**MP and LZ:** Collected the data and performed the analysis.

**MP:** Was involved in the writing of the manuscript and is responsible for the integrity of the study.

All authors have read and approved the final manuscript.
